# Analysis of the carbon effect of high-standard basic farmland based on the whole life cycle

**DOI:** 10.1038/s41598-024-53432-2

**Published:** 2024-02-09

**Authors:** Xuemei Li, Ying He, Yanhua Fu, Yajie Wang

**Affiliations:** College of Economics and Management, Tianjin Chengjian University, Tianjin, 300192 China

**Keywords:** Carbon cycle, Agroecology, Ecosystem ecology

## Abstract

Based on the whole life cycle theory, the carbon effect of three different sizes of high-standard basic farmland construction projects is measured and analysed. The results show that the carbon emissions generated during the construction of high-standard basic farmland projects and the carbon absorption capacity at the later stage are different for projects of different sizes. The carbon emissions of different scales of high-standard basic farmland projects will increase during the construction stage. The results of carbon effect changes in the later operation and management stage show that the high-standard basic farmland construction projects will help reduce the carbon emissions of the field ecosystem where the farmland is located and increase its carbon sink capacity after the completion of construction, which is more obvious in larger projects. The emission reduction and carbon sequestration capacity of the farmland after remediation are improved to different degrees, which is more conducive to the ecological development of agricultural production and ecological environmental protection in the relevant areas. The study contributes to the green development of farmland, which is of some significance for the sustainable development of agriculture in Tianjin and the whole country.

## Introduction

Agricultural activities are one of the main causes of global warming, and the key to effectively solving this problem is to reduce carbon emissions and increase carbon sequestration^1–3^. On the one hand, the total carbon emissions generated by agriculture are very large, accounting for approximately 25% of the total global carbon emissions^4^, and there is a long way to go to reduce carbon emissions. On the other hand, agricultural production activities, such as farmland and forests, can effectively absorb carbon dioxide and other greenhouse gases through their own carbon sequestration capacity, i.e., agriculture has a significant carbon sink capacity^5–7^.

Traditional farmland suffers from fragmented farming and poor infrastructure in the process of arable reclamation. This can result in significant emissions of greenhouse gases, such as carbon dioxide, which can cause varying degrees of damage to agricultural livelihoods and natural ecosystems. Ultimately, this affects the improvement of arable land quality and the reduction of carbon emissions from agriculture. The construction of high-standard basic farmland can effectively achieve a significant reduction in farmland carbon emissions and a stable increase in farmland carbon sequestration capacity through the implementation of land levelling projects, irrigation and drainage projects, field road projects, farmland protection projects and typical field remediation methods^8^. These practices could promote the further development of agricultural carbon emission reduction and sequestration. On May 7, 2022, the Ministry of Agriculture and Rural Affairs and National Development and Reform Commission passed the “Agricultural Rural Carbon Emission Reduction Program” and proposed that among the six key tasks and ten major actions for agricultural carbon emission reduction, the construction of high-standard basic farmland is an effective way to expand the carbon sequestration capacity of farmland, accelerate arable land governance and quickly compensate for the shortcomings of agricultural infrastructure.

Whole life cycle theory refers to a design concept that considers the entire construction cycle of a project at the initial design stage and plans all relevant factors in each stage of the construction cycle^9,10^. Currently, it is mostly used in various construction projects, i.e., the whole life cycle of engineering and construction projects, including the five stages of decision-making, design, construction, operation, and end-of-life recycling. The research results show that the carbon emissions generated by construction projects based on the whole life cycle theory are reduced, which is in line with the concept of green building development^11,12^.

The whole life cycle of the high-standard basic farmland construction process includes the decision design stage, construction stage and operation management stage from the beginning to the end of high-standard basic farmland construction^13^. The carbon effect is mainly reflected in the construction stage and operation management stage. Because the actual change in the carbon effect is not involved in the initial decision and design stage of the construction of high-standard basic farmland, this paper does not measure or analyse it. The carbon effect in the construction stage refers to the carbon emissions generated by the consumption of materials, the use of appliances and the input of personnel during the construction of high-standard basic farmland. For example, the use of different quantities of materials and energy, such as steel, cement and diesel, in the three selected projects is a carbon effect of the construction phase of the project. The carbon emissions and carbon absorption generated by the conversion of land-use types before and after the construction of the project are also part of the carbon effect in the construction stage^14^. The carbon effect in the operation and management stage refers to the change in the carbon effect in the field ecosystem due to the increase in effective arable land area and the improvement in land quality after the construction of high-standard basic farmland^15^.

From the point of view of existing research, China’s attention to the construction of high-standard farmland is focused on its feasibility and regional suitability research, and the analysis of the benefits of high-standard farmland construction projects is dominated by the analysis of economic, social and ecological benefits, and there is a lack of research on the whole lifecycle of the remediation process. Based on this, this paper focuses on the whole life cycle of high-standard farmland construction projects, combines the carbon emissions and carbon absorption (hereinafter referred to as the carbon effect) generated in the process of farmland remediation with it, takes different scales of remediation projects in Tianjin as an example, measures the carbon effect of the whole life cycle of farmland construction by using the relevant formulas, compares the changes in the carbon effect before and after the construction, and estimates the time to reach the carbon breakeven state after remediation of the farmland location, and summarizes the practical insights on the reduction of the carbon effect of the whole life cycle of the high standard farmland construction projects with the aim to promote the green development of the construction of high-standard farmland.

## Materials and methods

### Calculation method of the carbon effect in the engineering construction stage

#### Calculation of the carbon effect of engineering construction

During the construction of high-standard basic farmland, the large input of construction materials and the large amount of consumption of fossil and mechanical energy are the main sources of greenhouse gas emissions, which have a certain impact on the carbon balance of the regional ecosystem, and the carbon emissions of the project construction mainly come from the following aspects: (1) the typical field and land levelling construction process, with the use of a large number of agricultural machinery appliances, which consume fossil energy such as gasoline and diesel, causing carbon emissions; (2) the carbon emissions caused by the production and use of various materials such as bricks, steel and cement during the construction process; and (3) the carbon emissions indirectly caused by the input of a large number of workers during the construction process, which cannot be ignored. In this paper, the carbon emissions from the above three aspects are integrated and specifically measured by Eq. (1)^16^:

1 $$ {\text{C}}_{{\text{g}}} = \sum\limits_{{{\text{i}} = 1}}^{{\text{n}}} {\left( {{\text{M}}_{{\text{i}}} \times {\text{f}}_{{\text{i}}} } \right)} $$

where Cg denotes the total carbon emissions from construction; Mi denotes the carbon emissions caused by the above three aspects; and fi denotes the respective carbon emission coefficient. Among them, the carbon emission coefficients of various materials are compiled according to IPCC guidelines^17^ and relevant literature studies^18^, as shown in Table 1 below.

**Table 1:** Carbon emission factors for major materials, energy and personnel.

Steel reinforcement/(kg/kg)	Diesel/(kg/kg)	Gasoline/(kg/kg)	Electric power/(kg/kWh)
1.06	0.86	0.81	2.26

Stones/(kg/m^3^)	Standard tiles/(kg/1000 piece)	Cement/(kg/kg)	People{kg/(people.d)}
2.39	1452.3	0.136	18.90

#### Calculation of the carbon effect of landform conversion stage

After the high-standard basic farmland construction project, the abandoned grassland, uncultivated forestland and dirty water area in the construction area were effectively managed, and most of the fragmented land was centrally managed, which brought about the change in land use type, i.e., land type conversion, in the whole area, which affected the level of regional carbon emissions. In this paper, according to the existing research and the theories of related experts and scholars, the ecosystem type method was used to measure such carbon emissions, and the corresponding carbon emission changes were calculated by the conversion of land use types and the changes in soil carbon stocks before and after the completion of the high-standard basic farmland construction project (the surface vegetation was destroyed during the construction process of the project, so it was not included in the calculation process). The measurement is done through the change of carbon effect due to land use change before and after the remediation. The specific measurement formula is as follows in Eq. (2)^19^:

2 $$ {\text{C}}_{{\text{d}}} {\text{ = C}}_{{{\text{d}}\left( {{\text{after}}} \right)}} - {\text{C}}_{{{\text{d}}\left( {{\text{before}}} \right)}} = {\text{S}} \times {\text{P}} $$

where Cd denotes the carbon emission change value of each land class conversion; Cd_(after)_ denotes the carbon emission value after land class conversion; Cd_(before)_ denotes the carbon emission value before land class conversion; S denotes the area matrix of each land class conversion (as shown in Table 2 below); and P denotes the soil carbon stock change matrix of each land class^20^.

**Table 2:** Land class conversion carbon effect matrix (kg/m^2^).

Land use type	Arable land	Woodland	Garden plot	Construction land	Other land	Water area
Arable land	0	–	–	–	–	–
Woodland	4.83	0	–	–	–	–
Garden plot	0.34	− 4.49	0	–	–	–
Construction land	− 2.53	− 7.36	− 2.87	0	–	–
Other land	− 2.37	− 7.20	− 2.71	0.16	0	–
Water area	− 1.67	− 6.50	− 2.01	0.86	0.70	0

Positive values in the above table are carbon emissions and negative values are carbon sequestration.

### Calculation method of the carbon effect in the operation management stage

After the completion of the high-standard basic farmland construction project, the area and quality of the cultivated land in its area will be significantly improved, which will increase the unit yield and overall yield of the agricultural crops, thus enhancing the carbon sequestration capacity of the whole regional farmland ecosystem, i.e., the carbon absorption capacity of agricultural crops is greatly enhanced, which is also the main source of the change in the carbon effect in the operation and management stage in the construction of high-standard basic farmland. The carbon sequestration rate can be estimated with reference to its own average water content, economic coefficient, and carbon sequestration rate (Table 3 below), as shown in Eq. (3)^21^:

3 $$ {\text{C}}_{{\text{n}}} = \sum {{\text{C}}_{{{\text{i}}\left( {{\text{absorb}}} \right)}} = \sum {\left[ {\left( {1 - {\text{W}}_{{\text{i}}} } \right) \times \frac{1}{{{\text{H}}_{{\text{i}}} }} \times {\text{f}}_{{{\text{i}}\left( {{\text{absorb}}} \right)}} } \right]} } $$

where Cn indicates the total carbon uptake per unit agricultural crop (kg/kg); Ci absorb indicates the carbon uptake per unit agricultural crop of class i (kg/kg); Wi indicates the average water content of class i agricultural crops; Hi indicates the economic coefficient of class i agricultural crops; and fi absorb indicates the carbon uptake rate per unit agricultural crop (%).

**Table 3:** Carbon sequestration rate per unit of agricultural crop yield.

	Average moisture content (Wi)	Economic factor (Hi)	Absorption rate (fi(absorb))	Ci (absorb)
Wheat	0.130	0.400	49.000	1.065
Corn	0.130	0.400	47.100	1.024

### Carbon balance calculation

During the construction of high-standard basic farmland, a large amount of carbon emissions are often generated due to the large amount of land levelling and use of agricultural machinery. After the construction project is completed, the carbon absorption capacity will be greatly improved due to the improvement of arable land quality and agricultural production conditions. By combining the two effects, the total carbon absorption in the construction area will offset the carbon emissions generated during the construction process for a period of time after the construction of high-standard farmland is completed, reaching a state of carbon balance. Based on the above, this paper constructed a carbon profit and loss calculation model for the construction of high-standard basic farmland with reference to existing studies to determine the degree of impact of high-standard basic farmland construction projects on the carbon balance of their locations. The specific calculation model is shown in Eq. (4)^22^:

4 $$ {\text{Y}} = \left( {{\text{Cg}} - {\text{Cd}}} \right)/{\text{Cn}} $$

where Y indicates the time required for the site to reach carbon balance after the completion of the high-standard basic farmland construction project; Cg denotes the total carbon emissions from construction; C_d_ denotes the carbon emission change value of each land class conversion; C_n_ indicates the total carbon uptake per unit agricultural crop (kg/kg).

### Data sources

**Figure 1:**
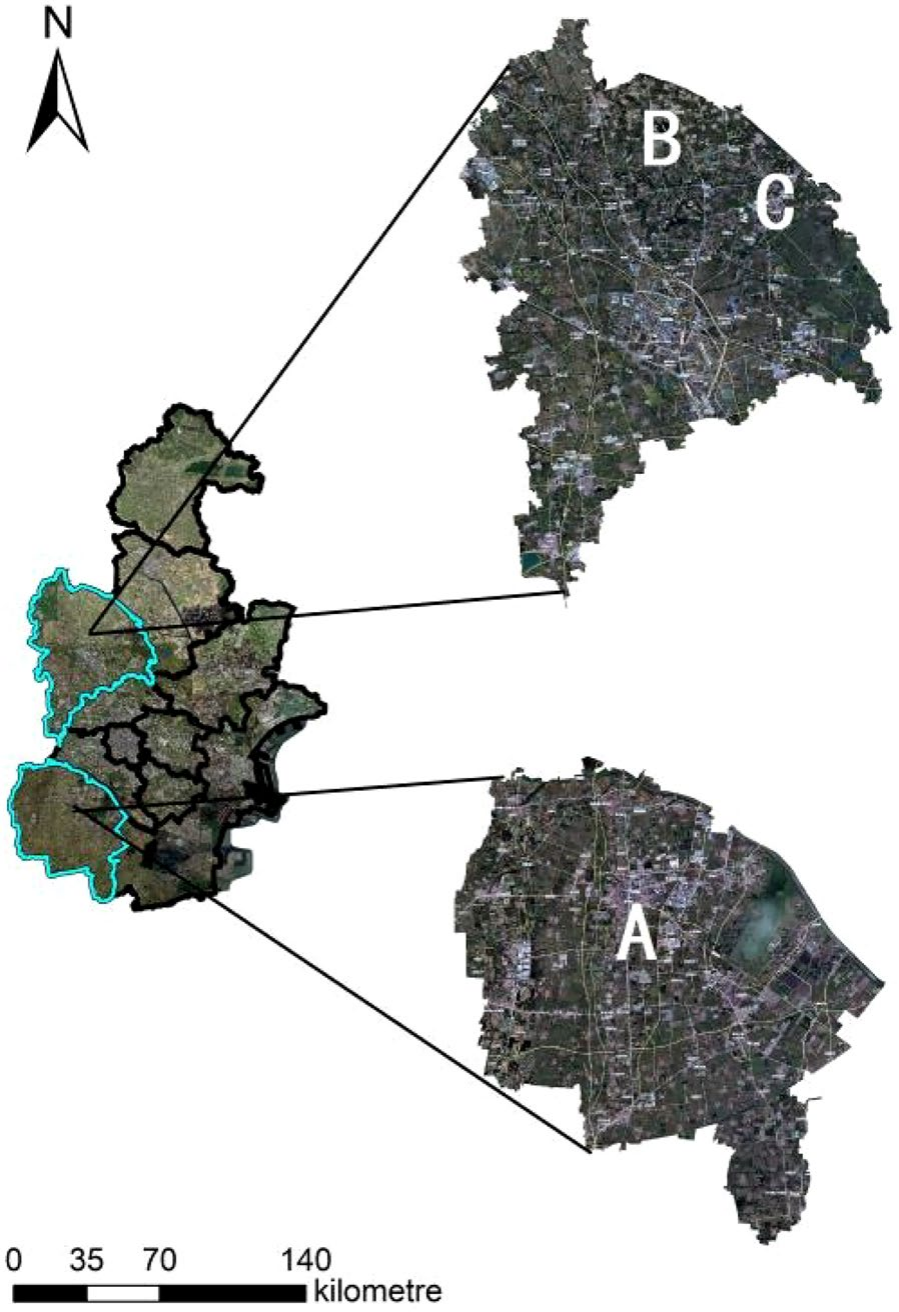
Schematic of the project area.

In this paper, three high-standard basic farmland construction projects of different scales in Tianjin, China, were selected as case studies (Project A, Project B and Project C). The three projects are located in different directions in Tianjin (as shown in Fig. 1). Project A is located in the northeast of Tianjin, with a low and flat terrain; the terrain belongs to the alluvial plain area, the topography is relatively gentle, the elevation is between 2 and 7 m, it is a typical low plain landform, and depressions and flat lands are the main types of land forms here. Project B is located in the north of Tianjin, with a gently sloping ground, approximately 6 m above sea level, little difference in elevation, an interwoven river network, and a vast area of blue. Project C is located in the east of Tianjin, with a gently sloping ground, with little difference in elevation, more depressions and deposits, and a vast area of openness. The three projects are located in the northern plain area, the terrain is relatively gentle, and the construction project difference is small; only the construction scale had a large difference. Therefore, by comparing the differences in carbon emissions and carbon absorption capacity enhancement produced by these three projects throughout their life cycle and predicting the time to reach carbon balance after remediation for each of the three projects of different scales, we explored effective ways to improve the green development of farmland agriculture in the future, and we provide some reference for the study of the whole life cycle of the construction of high-standard basic farmland.

The three selected projects are located in the plain area with little difference in terrain. Therefore, it is mainly analysed and measured using some basic data combined with the relevant formulae and the land use conversion matrix. The basic data used are mainly the area of the construction scale, the area of new arable land, the amount of petrol used, the amount of diesel used, the amount of cement and steel used, and the amount of carbon absorbed by planted crops, among other indicators. And combined with field visits to the project area to maximize the elimination of data differences caused by other natural factors. The project details are shown in Table 4. The underlying data for the selected projects are derived from the design planning report for the project.

**Table 4:** Main data of high-standard basic farmland construction case study.

	Project A	Project B	Project C
Total area/ha	261.958	1550.959	3462.937
Construction scale/ha	253.344	1542.307	3439.578
Total investment/million yuan	735.317	2637.192	5549.319
New arable land area/ha	21.162	5.572	44.874
Rate of new cultivated land/%	8.1	0.28	1.97
Gasoline usage/kg	1910.65	18,615.93	62.25
Diesel usage/kg	37,796.38	54,101.15	143,055.64
Project area landform type	Plain	Plain	Plain
Main crops grown	Corn, wheat	Corn, wheat	Corn, wheat

## Results

### Analysis of the carbon effect of the construction phase of the case studies

#### Carbon effect of engineering construction

**Figure 2:**
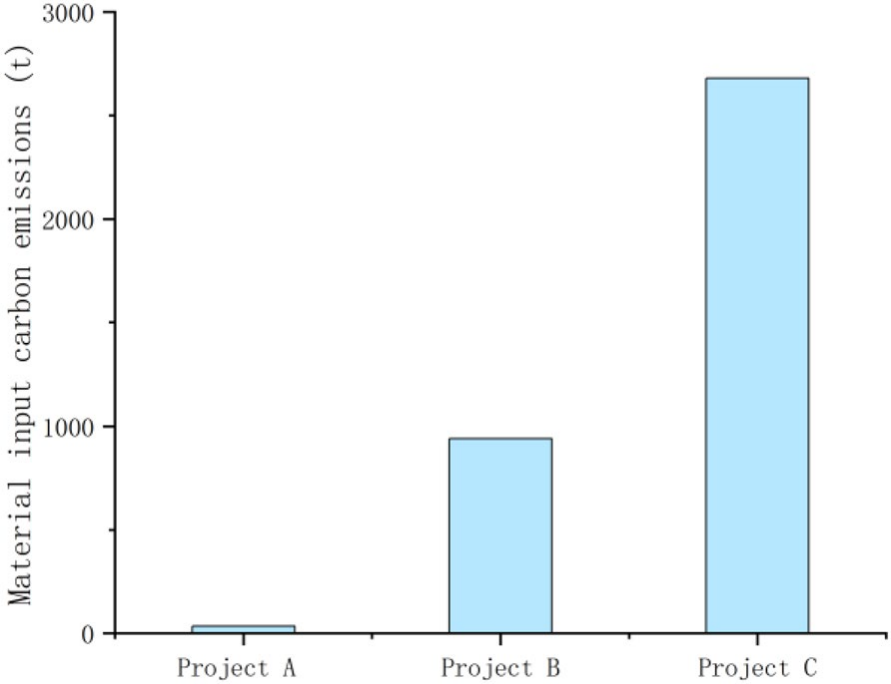
Carbon emissions from material inputs.

**Figure 3:**
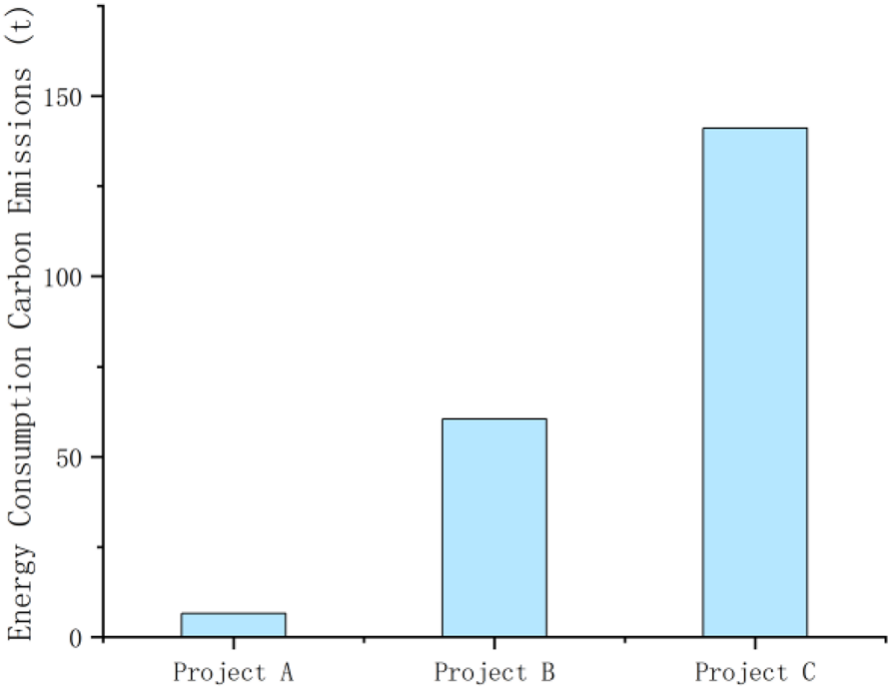
Carbon emissions from energy consumption.

**Figure 4:**
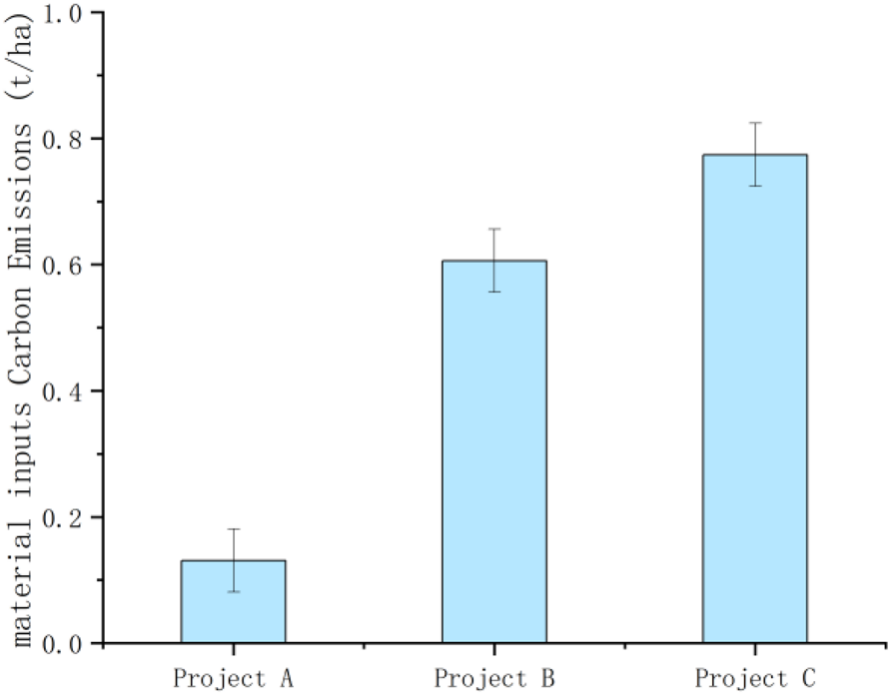
Carbon emissions from material inputs (per hectare).

**Figure 5:**
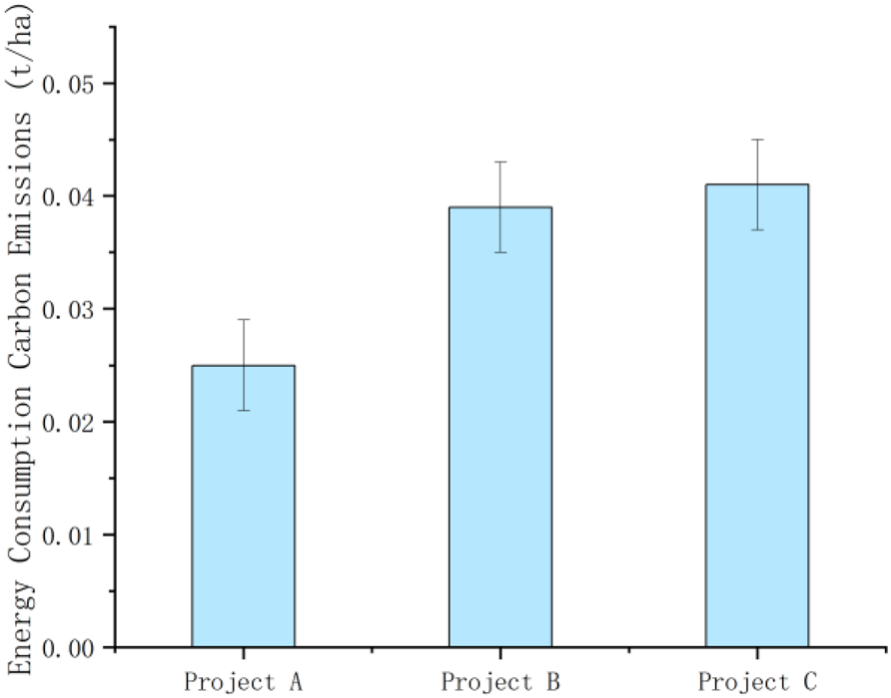
Carbon emissions from energy consumption (per hectare).

From Formula (1), it can be seen that the carbon emissions generated by the combination of the cement, steel, block, standard bricks, diesel and gasoline used in the construction of construction projects A, B and C with their respective carbon emission factors were 40.519 t, 1001.061 t and 2820.058 t, respectively. In terms of the carbon emissions from material inputs, the carbon emissions from cement were 27.526 t, 919.998 t and 2508.925 t, accounting for most of the total emissions; the carbon emissions from bricks were 1.251 t, 3.554 t and 0.428 t; the carbon emissions from standard bricks were 0.3265 t, 5.577 t and 130.344 t; the carbon emissions from steel bars were 0.668 t, 0.711 t and 2.034 t; and the carbon emissions from personnel were 4.548 t, 10.697 t and 37.254 t. In terms of the carbon emissions from energy consumption, diesel fuel accounted for the highest proportion of the three construction projects, with 4.974 t, 45.445 t, and 141.021 t, respectively, while gasoline emissions were 1.548 t, 15.079 t, and 0.051 t. As the projects selected in the study are high-standard farmland remediation projects of different scales, with large differences in area, and the study mainly compares the changes in carbon effects before and after the construction of the remediation project itself, the standard errors are not made in the overall material inputs and energy consumption graphs below, and the error lines and standard errors for the selected projects are in the carbon emissions per hectare produced by the three projects (As shown in Figs. 2, 3, 4, 5 below). As shown in the figure, the carbon emissions from material inputs per hectare for projects A, B and C during the construction phase of the project are 0.131 ± 0.050 t/ha, 0.606 ± 0.050 t/ha and 0.774 ± 0.050 t/ha, respectively; and the carbon emissions from energy consumption per hectare are 0.025 ± 0.004 t/ha, 0.039 ± 0.004 t/ha and 0.041 ± 0.004 t/ha, respectively (i.e. standard errors were 0.050 and 0.004). The carbon emissions from material inputs and energy consumption of the three projects are shown in Figs. 2 and 3, which show that although the construction of basic farmland at different scales causes carbon emissions to increase for a short period of time, the overall carbon emissions generated are small and will not cause greater damage to the ecological environment where the farmland is located^23^.

#### Carbon effect of landform conversion stage

In terms of the carbon effect of land conversion, it can be seen from Eq. (2) and the land conversion matrix that the arable land in the construction areas of the three selected high-standard farmland construction projects increased by 20.525 ha, 5.572 ha and 44.874 ha, respectively, with the new arable land coming from fragmented forestland, abandoned garden land, transportation land, abandoned water area and other land, among which the most obvious change was in the area of water area, with decreases of 16.135 ha, 1.077 ha and 37.596 ha, respectively (Tables 5, 6, 7). The source of the new arable land in the project areas differed slightly. That in Project A mainly came from garden land, transportation land and water, and the most obvious reduction was in water area. Project B increased the area of arable land by 5.572 ha. The remaining fragmented forestland, abandoned garden land, transportation land, abandoned water and other land areas in the construction area were reduced by varying degrees, and their areas were reduced by 0.020 ha, 1.310 ha, 2.921 ha, 1.077 ha and 0.242 ha, respectively. The main sources of new arable land for Project C are grassland, woodland, transport land, waters, parkland and other land, which covers the widest range of land types and the largest area of new arable land^24^.

**Table 5:** Project A land class conversion and carbon emission changes.

Land type	Arable land	Garden plot	Transportation land	Water area	Total
Area of change/ha	21.162	− 0.434	− 4.709	− 16.135	42.44
Changes in carbon emissions/t	− 36,324.708	− 147.735	10,915.425	24,954.643	− 602.375

**Table 6:** Project B land class conversion and carbon emission changes.

Land type	Arable land	Woodland	Transportation land	Water area	Garden plot	Other	Total
Area of change/ha	5.572	− 0.020	− 2.921	− 1.077	− 1.310	− 0.242	7.494
Changes in carbon emissions/t	− 9564.369	− 100.657	7391.926	1799.207	− 445.498	574.132	− 345.258

**Table 7:** Project C land class conversion and carbon emission changes.

Land type	Arable land	Grassland	Woodland	Transportation land	Water area	Garden plot	Other	Total
Area of change/ha	44.874	− 0.332	− 5.811	1.836	− 37.596	− 2.511	− 0.463	93.423
Changes in carbon emissions/t	− 77,026.196	24,398.425	− 29,244.834	4647.778	62,807.008	− 853.435	1097.328	− 14,173.926

After farmland remediation, the carbon emissions of the three project areas were finally converted to negative values after land use conversion, indicating that the carbon emissions of the farmland sites were reduced and the carbon sink capacity of the land increased after the remediation of high-standard farmland construction projects of different scales, which was conducive to the accelerated green development of the farmland sites and the promotion of the regional sustainable development process.

### Analysis of the carbon effect in the operation and management phase of the case

**Figure 6:**
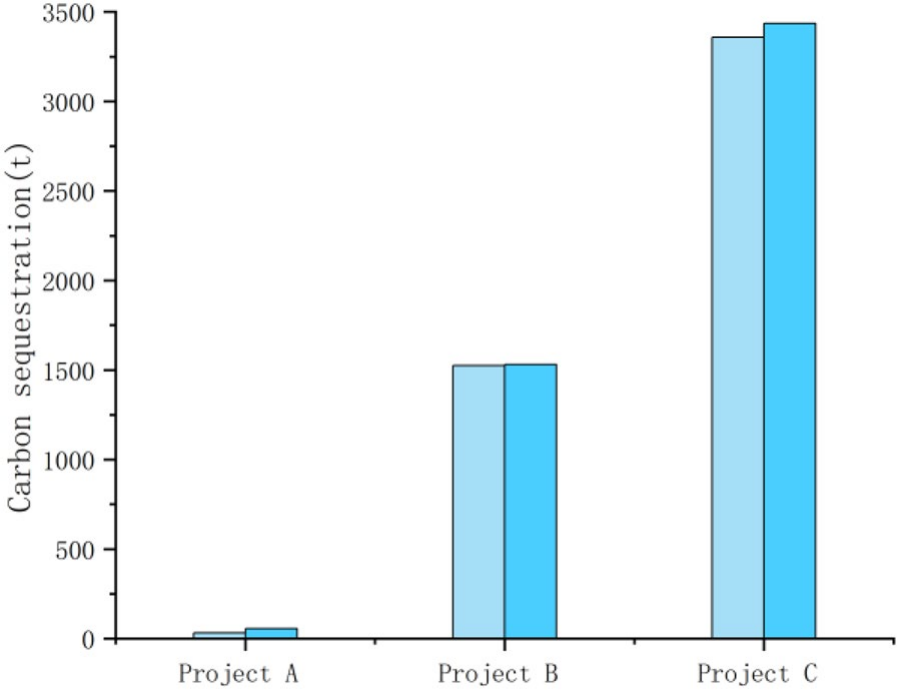
Carbon sequestration capacity of wheat before and after remediation.

**Figure 7:**
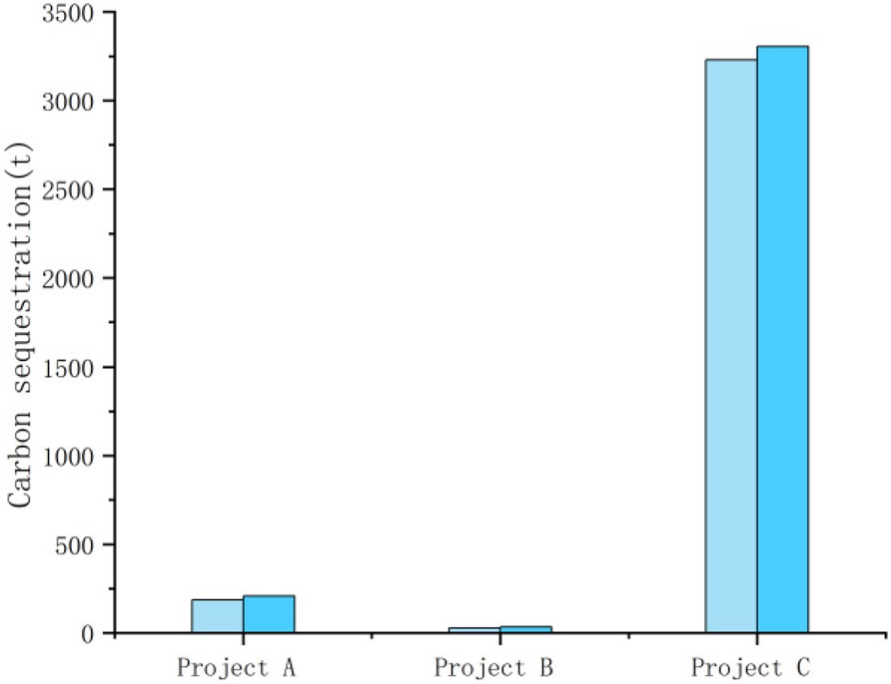
Carbon sequestration capacity of corn before and after remediation.

**Figure 8:**
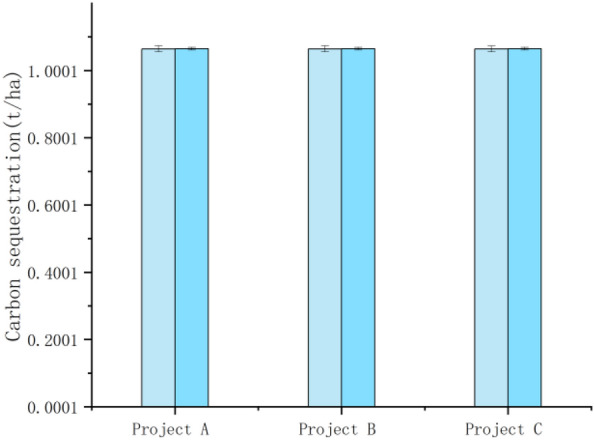
Wheat before and after remediation (per hectare).

**Figure 9:**
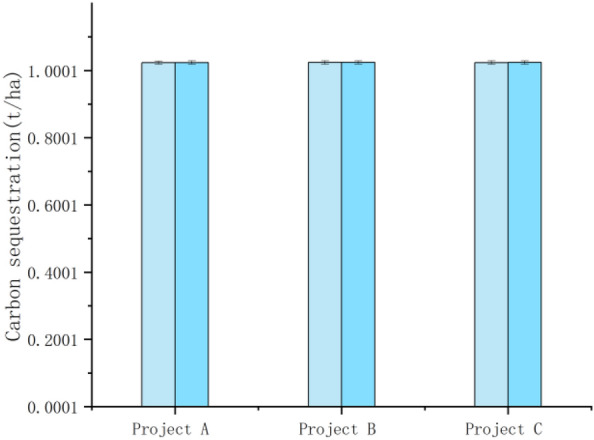
Corn before and after remediation (per hectare).

In terms of the carbon effect in the operation and management stage, the three cases had different area scales, but the main crops planted are corn and wheat. Therefore, through Eq. (3) and combined with the average water absorption rate and carbon absorption rate of corn and wheat planted in the three selected project sites of A, B and C, it can be seen that the carbon absorption capacity of the farmland ecosystem has increased, and the carbon sink capacity of the crops themselves has been enhanced to different degrees. The post-remediation measurements for the selected projects are all based on one year after completion of the construction project. Taking the carbon absorption capacity of these two crops as reference, the carbon absorption capacity of wheat and corn before remediation were 32.749 t and 187.321 t, 1525.356 t and 27.571 t, and 3357.619 t and 3228.359 t, respectively; after remediation, the carbon absorption capacity of wheat increased to 55.286 t, 1531.291 t and 3436.101 t, and that of maize increased to 208.991 t, 33.277 t and 3303.819 t, respectively. Due to the large difference in the scale of the selected projects and the large difference in the area of crops planted, instead of making standard errors on the graphs of changes in the overall carbon sequestration capacity of crops before and after construction, standard errors were made on the graphs of changes in the carbon sequestration capacity of crops per hectare before and after construction (Figs. 6, 7, 8, 9 below). As shown in the figure, the carbon sequestration capacity per hectare before remediation of the three selected projects was 1.0649 ± 0.0009 t/ha, 1.0647 ± 0.0009 t/ha, and 1.0645 ± 0.0009 t/ha for wheat, and 1.0237 ± 0.0005 t/ha, 1.0241 ± 0.0005 t/ha, and 1.0238 ± 0.0005 t/ha for corn (i.e., the standard errors before remediation were 0.0009 and 0.0005) . The increase in carbon sequestration capacity per hectare after remediation was 1.0651 ± 0.0004 t/ha, 1.0652 ± 0.0004 t/ha and 1.0650 ± 0.0004 t/ha for wheat, and 1.0239 ± 0.0005 t/ha, 1.0243 ± 0.0005 t/ha and 1.0242 ± 0.0005 t/ha for corn (i.e., the standard errors after remediation were 0.0004 and 0.0005). This change indicating that after the completion of the high-standard basic farmland construction project, with the farmland planting conditions greatly improved, the supporting infrastructure construction of the farmland tended to be perfected, and the growth conditions of crops were gradually optimized.

Equation (4) and the results of the carbon effect changes at each stage during the whole construction process of high-standard basic farmland were assessed after the completion of the construction of high-standard basic farmland.The three project sites A, B and C reached carbon balance after about 21 days, 96 days and 154 days, respectively, indicating that the ecosystems of different scales of high-standard basic farmland construction projects at the farmland site reached carbon break-even in different times after remediation^25^.

## Discussion

1. Reduce energy consumption in the construction stage of high-standard basic farmland construction and reduce carbon emission pressure in the construction stage^26,27^. Although carbon emissions will increase in the short term during the construction of the farmland site, after the completion of the construction, the quality of the farmland will be improved, the broken land will be more contiguous, the infrastructure will be gradually improved, and the environment for crop growing will be greatly improved, which effectively promotes the carbon balance of the local cropland ecosystem, and facilitates the efficient and green development of the farmland^28^. The construction of the farmland site is expected to be completed by the end of the year. In the construction process of high-standard basic farmland construction, the carbon effects brought by different remediation stages have differences, among which the engineering construction stage has the greatest impact on the total carbon emissions^29^. From the viewpoint of the material and personnel inputs, cement, steel and diesel were the main sources causing the increases in carbon emissions in the construction process, while protection projects such as farmland protection and field remediation reduced the pressure of carbon emissions to a certain extent^30,31^. The two had a certain balancing effect on the carbon effect at the project site^32^. Therefore, during the construction of high-standard basic farmland, the actual local situation should be fully integrated, and the production conditions of the farmland location should be carefully considered to reduce the use of large amounts of fossil energy as much as possible, reduce energy consumption, achieve ecological high-standard basic farmland construction, and promote the improvement of the regional farmland ecosystem and ecological environment^33^.
2. Scientific planning of the construction scale of high-standard basic farmland construction projects^34^. Scientific planning is needed to increase the carbon sink capacity of agricultural land parcels and improve their greenhouse gas absorption capacity^35^. To realize the ecological development of high-standard basic farmland construction at different scales and to prevent construction projects from failing to achieve the expected ecological development of high-standard basic farmland construction due to the unreasonable division between the scale and the area of the farmland or the ecological development of projects of different scales that are not in line with local policies^36^, the scale of high-standard basic farmland construction should be fully examined based on the actual situation of the area where the farmland is located. By fully investigating the actual situation of the area where the farmland is located, the high-standard basic farmland construction project should be planned on a reasonable scale and implemented effectively to achieve the organic unity of ecological, economic and social benefits in the construction process and provide an effective method for global carbon emission reduction^37^.
3. The factor of land type conversion should be fully considered to enhance the carbon sink capacity of land. In the process of high-standard basic farmland construction, land use type transformation, i.e., land class conversion, is essential, and this is one of the main ways to improve the carbon sink capacity of farmland sites^38^. In the process of land use conversion, the quality of cultivated land and crop production conditions are improved to a large extent, and the carbon emissions generated by farming are greatly reduced, which causes a significant increase in the overall carbon sink capacity of farmland^39,40^. Therefore, in the high-standard basic farmland construction process, the conversion factor of land types should be fully considered, and it should be closely integrated with the remediation project to guide local farmers to strengthen their awareness of ecological protection. The use of arable land should be adjusted to use less chemical and other fertilizers to further enhance the carbon sink capacity of the land^41^.

## Conclusions

By accounting for the carbon effects of the whole life cycle of the remediation process of high-standard basic farmland construction projects of different scales (including the carbon effects of the construction stage and the carbon effects of the operation and management stage), we systematically and comprehensively analysed the suppression effect of high-standard basic farmland construction of different scales on the carbon emissions of their locations and the level of improvement of their ecological environment; we drew the following conclusions by comparing the differences in the reduction of carbon emissions and the enhancement of the carbon absorption capacity of the construction projects of different scales, combined with the improvement of the carbon balance quality of the local farmland ecosystems:

1. The construction stage was the main reason for the rapid increase in the total carbon emissions of farmland. In the whole life cycle of the construction of high-standard basic farmland projects, the construction stage was the main reason for the rapid increase in the total carbon emissions in farmland in the short term, among which the use of cement and diesel were the main reasons. The total amount of carbon emissions in the short term was larger in larger projects, while the total amount of carbon emissions in the short term was smaller in smaller projects.
2. The carbon effect generated by different scales of construction and land conversion types differed. In the process of construction of high-standard basic farmland of different scales, the carbon effect of engineering construction tended to increase because of land levelling, irrigation and drainage, and farmland protection. The process of land conversion will produce different degrees of carbon dioxide and other greenhouse gas emissions, and the carbon effects of different engineering construction and land conversion types are different.
3. The construction of high-standard basic farmland will lead to a decrease in the carbon effect. Although in the short term, the construction of high-standard basic farmland will increase the carbon effect at each location, the carbon effect will reach a carbon equilibrium state after a short period of growth and then gradually decline because the quality of farmland is greatly improved and the cropping conditions are greatly improved. Therefore, in the long run, the construction of high-standard basic farmland will contribute to a decrease in the local carbon effect.
4. The construction of high-standard basic farmland will increase the carbon stock of farmland. As the quality of arable land improves, its carbon storage subsequently increases, which is the main reason why the total carbon storage of the farmland location increases greatly after the construction of high-standard basic farmland, while the reductions of forestland, garden land, agricultural transportation land and water area to different degrees in the process of land type conversion of plain-type construction projects were key factors in the reduction of soil carbon storage and carbon emissions.

## Data Availability

The datasets used and/or analysed during the current study available from the corresponding author on reasonable request.
